# Mental health crisis resolution teams and crisis care systems in England: a national survey

**DOI:** 10.1192/bjb.2018.19

**Published:** 2018-08

**Authors:** Brynmor Lloyd-Evans, Danielle Lamb, Joseph Barnby, Michelle Eskinazi, Amelia Turner, Sonia Johnson

**Affiliations:** 1 University College London

## Abstract

**Aims and method:**

A national survey investigated the implementation of mental health crisis resolution teams (CRTs) in England. CRTs were mapped and team managers completed an online survey.

**Results:**

Ninety-five per cent of mapped CRTs (*n* = 233) completed the survey. Few CRTs adhered fully to national policy guidelines. CRT implementation and local acute care system contexts varied substantially. Access to CRTs for working-age adults appears to have improved, compared with a similar survey in 2012, despite no evidence of higher staffing levels. Specialist CRTs for children and for older adults with dementia have been implemented in some areas but are uncommon.

**Clinical implications:**

A national mandate and policy guidelines have been insufficient to implement CRTs fully as planned. Programmes to support adherence to the CRT model and CRT service improvement are required. Clearer policy guidance is needed on requirements for crisis care for young people and older adults.

**Declaration of interest:**

None.

Crisis resolution teams (CRTs) are multidisciplinary, specialist mental health services that offer brief intensive home treatment to people experiencing a mental health crisis, with the aim of averting hospital admission wherever possible.^1^ CRTs for working-age adults have been implemented nationally in England since the National Health Service (NHS) Plan of 2000,^2^ and elsewhere in Europe and Australasia.^3^ The English national mandate for CRTs was accompanied by policy implementation guidance^4^; the CRT model it specified has been endorsed in numerous subsequent policy guidelines.^5^ Trials suggest that CRTs can reduce in-patient admissions and increase patients' satisfaction with acute care.^6^^,^^7^ However, when scaled up to national level, the implementation of CRTs has been highly variable,^8^^–^^10^ and their effects on admission rates have been disappointing.^11^ In England, improving access to and quality of mental health crisis care across the age range has been identified as a priority by expert bodies and policy makers.^12^^–^^15^ To inform future mental health workforce planning, in 2016 Health Education England commissioned a team from University College London to conduct a national survey of CRTs.

## Method

### Aims

The nationwide implementation of CRTs for working age adults in England mandated by the NHS Plan^2^ represents an unusually prescriptive attempt to implement a new mental health service model on a national scale. In this paper, we aimed to investigate the consequences of this national implementation, through addressing two main research questions. First, to what extent do CRTs adhere to the implementation guidance for CRTs^4^ that accompanied the national mandate? Second, how has the implementation of CRTs changed, compared with results from a similar CRT survey^9^ conducted in 2012? Secondary aims were to map the provision of CRTs in England for working-age adults, children, and older adults and people with dementia; to explore variation in the local acute care system contexts in which CRTs operate; and to describe the staffing and access arrangements of CRTs.

### Setting

We sought to map and include all CRTs in England. CRTs were defined as mental health services that exclusively provided brief, intensive home treatment for people in mental health crisis with the aim of averting hospital admission. Services that provided longer-term intensive home treatment (e.g. assertive community treatment teams) or intensive home treatment as part of a broader community service (e.g. within the context of a community mental health team) were excluded.

### Participants

Team managers of each identified CRT were invited to participate in the survey. Where the manager was unavailable, an alternative senior CRT staff respondent was sought.

### Measures

The study team developed a 91-item questionnaire, informed by the measure used in a previous CRT survey.^9^ This included a mixture of quantitative questions and questions requiring brief free-text responses. Questions covered: current CRT staffing and caseload size; referral and access arrangements, including opening hours, eligibility criteria, referral routes and response time; working arrangements with other acute services; the role of the CRT in decision-making regarding hospital admission (‘gatekeeping’); staff training; and team philosophy of care.

### Procedures

CRTs were mapped by multiple means, including registers of adult and older adult CRTs from two research studies and a national quality improvement network, and through the websites of all mental health NHS trusts in England. CRT managers were asked to identify other CRTs in their area (including teams for young people or older adults) when contacted about the survey, in order to identify any CRTs previously missed.

The survey met Health Research Authority criteria for a service evaluation^16^ and was approved as such by the North London Research Consortium. Local processes for approving the service evaluation were followed by the study team wherever required.

Details of mapped CRTs were entered into Opinio,^17^ UCL's secure online survey system. Team managers were then automatically invited to complete the survey by email, through Opinio, and were also contacted by researchers, sent an information sheet about the study and given the opportunity to ask any questions. Following three, weekly automatic Opinio email reminders, researchers contacted remaining non-responders by phone. The option of completing the questionnaire as a telephone interview was offered. Respondents consented to take part by completing the survey: those who completed the survey online entered their own data directly into Opinio; researchers entered the data into Opinio for phone respondents.

The survey took place from September to November 2016. At the beginning of December 2016, the online survey was closed and the data were downloaded from Opinio into SPSS for Windows for data analysis. Data files were stored on the secure, password-protected UCL IT system.

### Analysis

Data were analysed in SPSS and descriptive statistics were presented separately for adult, older adult/dementia, and children and young people's CRT teams. Survey questions which directly mapped on to national policy implementation guidance for adult CRTs^4^ were identified, and the proportion of adult CRTs meeting each policy recommendation was reported. For variables where comparable data were available from both our survey and the 2012 national CRT survey,^9^ differences in responses between the two time points were explored using bivariate statistics.

## Results

Overall, 198 adult CRTs, 15 CRTs for children and young people, and 31 CRTs for older adults and/or people with dementia were mapped. (One adult CRT originally mapped in error was excluded.) Survey responses were obtained from 190 adult teams, 13 children and young people's teams, and 30 older adult teams: an overall response rate of 95%. Two administrative health regions (NHS trusts) had no adult CRT services: in these regions, crisis response was organised within broader community mental health teams.

### Adult CRTs' adherence to policy recommendations

Table 1 summarises how far adult CRTs were adhering to the recommendations of the influential Mental Health Policy Implementation Guide^4^ which accompanied the national mandate for adult CRTs in England. Only one team was fully adherent in all respects; recommendations for having a multidisciplinary staff team and for accepting referrals directly from general practitioners (GPs) and patients were most frequently unmet. Regarding staffing levels, 76% of teams met the minimum recommended staffing level of 14 full time equivalent staff for a caseload of 30 patients, based on their current caseload on the day of the survey. However, this figure dropped to 55% of teams, based on their reported highest typical caseload.

**Table 1:** Adult CRTs' adherence to national policy implementation guidance regarding access and staffing

Department of Health 2001 policy implementation guidance requirement for CRTs	Proportion of CRTs for working age adults implementing this guidance *n*/*N* (%)
The CRT can provide home treatment 24 h a day, 7 days a week [Coded as: the CRT can provide home visits to patients on its caseload at any time of the day or night]	132/190 (70%)
The CRT has easy referral processes including accepting direct referral from GPs and patients/families	78/185 (42%)
The CRT will work with adults aged 16–65 years	42/190 (22%)
The CRT should act as gatekeeper to in-patient services [Coded as: does the CRT always assess voluntary patients in person before hospital admission?]	92/185 (50%)
Adherence to all the above access requirements	33/185 (18%)
The CRT includes a psychiatrist [Coded as: the CRT includes a consultant or staff grade psychiatrist]	173/185 (94%)
The CRT team should be multidisciplinary [Coded as: the CRT includes psychiatrist, nursing, social work, psychologist and occupational therapist staff and support workers]	27/185 (15%)
The CRT should include at least 14 full time equivalent staff for a team caseload of up to 30 patients [Coded based on current caseload from survey responses]	137/180 (76%)
Adherence to all the above staffing requirements	17/180 (9%)
Adherence to all staffing and access requirements	1/180 (1%)

### Changes to CRT implementation 2012–2016

Table 2 summarises recent changes in adult CRT implementation, based on a comparison of results from this survey with those from a previous national survey conducted in 2012.^9^ Overall, adult CRTs appear to be more accessible in 2016: compared with 2012, significantly more CRTs accept self-referrals, provide a 24 h service, and work with people with personality disorders and with older adults. An exception to this trend is that fewer CRTs in 2016 accepted young people aged 16–17 years. More CRTs in 2016 were able to access non-hospital crisis beds, compared with 2012, and more teams in 2016 fulfilled a full ‘gatekeeping' function and assessed all patients before voluntary hospital admission. Changes in CRTs' staff mix were less marked, although social workers were less well represented and psychologists were better represented in CRTs in 2016 than in 2012. The proportion of teams meeting recommended minimum staffing levels fell from 87 to 76%.

**Table 2:** Implementation of CRTs for working age adults in 2016 compared with 2012

Service domain	CRT characteristic	CRTs with this service characteristic *n*/*N* (%)		Significant differences 2012–2016
		2012 CRT survey	2016 CRT survey	
Eligibility (diagnosis)	The CRT will accept people with dementia	39/192 (20%)	32/190 (17%)	n/s
	The CRT will accept people with comorbid learning difficulties	111/192 (58%)	94/190 (50%)	n/s
	The CRT will accept people with personality disorder	151/192 (79%)	187/190 (98%)	χ^2^ = 36.6, *P* < 0.001
Eligibility (age)	The CRT will accept people age 16+	99/192 (52%)	60/190 (32%)	χ^2^ = 15.7, *P* < 0.001
	There is no upper age limit to the CRT service	110/191 (58%)	137/190 (72%)	χ^2^ = 8.8, *P* = 0.003
Access (hours of service)	The CRT provides a 24 h telephone response	138/171 (81%)	176/190 (93%)	χ^2^ = 11.3, *P* = 0.001
	The CRT provides home visits 24/7	65/166 (39%)	132/190 (69%)	χ^2^ = 32.9, *P* < 0.001
Access (referrals)	The CRT accepts referrals from GPs	147/190 (77%)	148/184 (80%)	n/s
	The CRT accepts self-referrals from known patients	106/191 (55%)	127/184 (69%)	χ^2^ = 7.4, *P* = 0.007
	The CRT accepts self-referrals from new patients	40/191 (20.9%)	79/184 (43%)	χ^2^ = 20.9, *P* < 0.001
Access (gatekeeping)	The CRT assesses all patients in person before voluntary hospital admission	62/187 (33%)	92/185 (50%)	χ^2^ = 10.5, *P* = 0.001
	The CRT always attends Mental Health Act assessments	35/187 (19%)	35/185 (19%)	n/s
Staff mix	The CRT includes consultant psychiatrists	148/171 (87%)	163/185 (88%)	n/s
	The CRT includes psychiatrists at non-consultant grades	129/171 (75%)	133/185 (72%)	n/s
	The CRT includes nurses	171/171 (100%)	182/185 (98%)	n/s
	The CRT includes social workers	122/171 (71%)	105/185 (57%)	χ^2^ = 8.2, *P* = 0.004
	The CRT includes occupational therapists	72/171 (42%)	88/185 (48%)	n/s
	The CRT includes psychologists	50/171 (29%)	73/185 (39%)	χ^2^ = 4.1, *P* = 0.04
	The CRT includes support workers	145/171 (85%)	162/185 (88%)	n/s
Staffing level	The CRT has at least 14 full time equivalent staff for a caseload of 30 patients (based on current caseload)	116/134 (87%)	137/180 (76%)	χ^2^ = 5.4, *P* = 0.02
Crisis alternatives	The CRT has access to a crisis house	65/184 (35%)	85/185 (46%)	χ^2^ = 4.3, *P* = 0.04
	The CRT has access to an acute day hospital	41/184 (22%)	40/185 (22%)	n/s

### CRTs within the acute care system

Table 3 shows the different acute care contexts within which CRTs operate. While all NHS trusts include acute in-patient wards, there was wide variation in the availability of other crisis services within local acute care systems. About half of adult CRTs were supported by a separate, staffed crisis phone line, and had access to residential, non-hospital crisis beds. About one-fifth of adult CRTs could access places for patients at an acute day hospital. Three innovations in acute care systems are highlighted by the survey. First, there is a split between crisis assessment and crisis home treatment functions: nearly one-third of adult CRTs are now supported by a separate triage/crisis assessment service. Second, 15% of adult CRTs are supported by non-residential crisis drop-in services, which typically function at evenings and on weekends, and can signpost elsewhere or refer individuals to CRTs. Third, specialist CRTs for young people and older adults, which were not nationally mandated, have been developed: these typically have less access than adult CRTs to other supportive crisis services.

**Table 3:** Variation in the acute care systems within which CRTs operate

Acute care system characteristic	CRTs operating within this type of acute care system n/N (%)		
	Adult CRTs	Children and young people's CRTs	Older adult/ dementia CRTs
A separate, 24 h crisis line is provided	106/184 (58%)	3/13 (23%)	10/29 (34%)
A separate crisis assessment/triage service is provided	59/184 (32%)	2/13 (15%)	6/29 (21%)
The CRT has access to residential crisis beds (non-hospital)	85/185 (46%)	1/13 (8%)	3/29 (10%)
The CRT can access an acute day hospital	40/185 (22%)	1/13 (8%)	5/29 (17%)
A separate sanctuary/crisis drop-in service is provided	28/185 (15%)	1/13 (8%)	3/29 (10%)

### Staffing and access: adult CRTs

Full descriptive results from the survey are provided in the supplementary data (File DS1) available at https://doi.org/10.1192/bjb.2018.19. Adult CRTs exhibited wide variation in staffing and access arrangements. While most teams included nurses (98%), psychiatrists (94%) and support workers (88%), whether teams included social workers (57%), occupational therapists (48%) or psychologists (39%) was much more variable. Current team caseloads varied from 5 to 144 patients; current staffing varied from 3 to 69 full time equivalent staff. A typical adult CRT, based on median scores, comprised 21 full time staff for a caseload of 29 patients.

Eligibility criteria for adult CRTs also varied. Most teams (72%) accepted patients over the age of 18 with no upper age limit, but only a third of teams (32%) would support young people aged 16–17. This was in the context of 13% of adult CRTs reporting that there was a local children and young people's CRT which operated 24 h a day, and 6% of adult CRTs with a local older adults' CRT operating 24 h a day. Half of adult CRTs (50%) reported that they would accept patients with comorbid learning difficulties, and only a minority (17%) supported people with dementia. Referrals from GPs were accepted by 80% of teams; self-referrals were accepted by two-thirds of teams (69%) if the patient was already known to services, but by fewer than half (43%) if the person was not previously known.

Most teams (93%) provided a 24 h telephone response, but just over two-thirds (69%) operated a full 24 h service, including capacity to make home visits. Half of adult CRTs (50%) reported that they always assessed patients in person before hospital admission was arranged, but only 19% of teams reported always attending Mental Health Act assessments, which precede compulsory hospital admissions. Eighty-six per cent of adult CRTs set a target response time for starting an assessment, having accepted a referral for a patient in crisis, but these targets varied from 1 h to 1 week. In 45% of teams, this target response time was 4 h or less.

### Staffing and access: children and young people's and older adult CRTs

While nurses were represented in all teams, only a minority of children and young people's CRTs (46%) and older adult CRTs (38%) included medical staffing. Occupational therapists were included in a majority of older adult teams (55%), and social workers in a majority of children and young people's teams (61%). For children and young people's teams, current caseload size ranged from 3 to 49 patients, and 59% of teams met a minimum staffing level benchmark of 14 full time equivalent staff for a caseload of 30 patients. For older adults, current caseloads ranged from 8 to 226 patients; 59% of these teams also met the minimum staffing level.

All but two of the children and young people's CRTs accepted all ages up to 18 years the other two had lower limits of 11 and 12 years, respectively. Of the 30 older adult teams included in the survey, 11 were exclusively for people with dementia, while the other 19 also accepted older adults with mental illness. Compared with adult CRTs, fewer CRTs for older adults (30%) and for children and young people (46%) offered a full 24 h service, including capacity to provide home visits. While most teams would accept direct referrals from GPs (69% of children and young people's teams; 76% of older adult teams), fewer than half would accept any referrals directly from patients or their families (46% for people already known to services in children and young people's CRTs; 45% in older adult CRTs). Target response times for starting an assessment following a new referral were very varied, as in adult CRTs: the response time target was 4 h or less for 64% of children and young people's teams which set a target, and for 33% of older adult teams. Only about a third of older adult and children and young people's teams (31% for each) reported always assessing patients in person before hospital admission.

### Philosophy of care and staff training

Forty-four per cent of adult CRTs and 52% of older adult CRTs reported having any philosophy of care or theoretical model which underpinned their service, with the recovery model being by far the most common response in each case. Only three of 13 children and young people's CRTs reported any underpinning philosophy or model – either a ‘psychosocial' model or a dialectical behaviour therapy approach. Fewer than half of CRTs for adults (41%), older adults (28%), or children and young people (31%) reported providing any CRT-specific training for the whole staff team.

## Discussion

The survey findings show that current implementation of the CRT model is highly variable. Almost no adult CRTs adhere fully to the model recommended in policy guidance. This is consistent with the findings from previous surveys.^8^^,^^9^ Adult CRTs appear to have become more accessible since 2012. The finding that fewer adult CRTs met recommended staffing levels in 2016 compared with 2012 should be treated with caution: it may be an artefact of a better response rate to the relevant questions in the 2016 survey, and may also reflect the proliferation of separate crisis assessment services, which, where present, reduce the workload for CRTs in responding to new referrals.

While adult CRTs remain almost universal in England, CRTs for children and for older adults are comparatively rare. The teams which do exist may serve larger geographical areas than adult CRTs, but in most areas of England, neither children nor adults with dementia can access crisis support from a specialist CRT team. Children's and older adult CRTs are typically less well staffed and less likely to be organised to provide easy-access, 24 h intensive home treatment, compared to adult CRTs.

### Strengths and limitations

The very high response rate provides confidence that this survey is representative of CRTs in England. As a self-report questionnaire, it is vulnerable to social desirability bias and to the possibility that respondents do not all interpret questions in the same way. It provides only a cross-sectional snapshot of CRT implementation at one moment in late 2016, although the comparison with results from a similar survey in 2012 allow some assessment of changes over time. Our survey did not ask about the types of intervention provided by CRTs.

### Implications for research

Four priorities for future research can be identified from this service evaluation. First, there is a need to evaluate mental health crisis care systems, not just individual service models. Table 3 showed that CRTs are operating in extremely variable acute service contexts; these contexts – both the configuration of crisis services and the continuity of care among them – are likely to influence outcomes for CRT patients and the overall effectiveness and costs of acute care. The separation of crisis assessment and home treatment teams in many areas represents a major change in acute care in England, which appears to have occurred in response to perceived local need rather than policy guidance or supporting research evidence. We lack evidence about optimal acute service system models. Second, a systematic review^18^ has highlighted the lack of high-quality evidence regarding older adult CRTs, and evidence is equally lacking for effective models of crisis care for younger people. It is unclear whether an adult CRT model is also appropriate for these client groups, or how the model should differ: specification and evaluation of CRT service models for children and older adults is required. Third, our survey suggests that CRTs may be improving access to care without increased staffing resources. This may be occurring in the context of increased demand for CRT services and reduced budgets.^19^ The effects of absorbing these pressures on the quality of care delivered to CRT patients are unknown. The need to understand the relationships between CRT resources, service organisation and access, and the quality of care provided to patients is therefore of high importance. A recently developed fidelity measure for CRTs^20^ offers a means to assess the organisation and delivery of care in CRT services rigorously and reliably, which could help to address this need. Fourth, the lack of adherence to best practice recommendations in adult CRTs indicates a need to develop resources to support CRTs in achieving high model fidelity and service quality. A current nationally funded study^21^ is evaluating a service improvement programme for CRTs in a cluster randomised trial, which, if effective, should help to address this need.

### Implications for practice

A generalisable implication of this service evaluation is that a policy mandate and guidelines are insufficient to achieve complete and consistent implementation of a desired service model. Active monitoring and support has been shown to be essential for the successful implementation of complex interventions in mental health in international contexts.^22^ Clear specification of desired service standards for CRTs, with audit and service improvement support to identify and address difficulties with implementation, is required for CRTs at local and national levels. For example, there is a huge difference for someone in a mental health crisis between waiting an hour for CRT support and waiting a week – yet this is the range of local response time targets reported by CRTs. This survey provides benchmarking data, which can inform the setting of feasible national standards for CRTs and assessment of future changes in CRT implementation. The apparent recent improvements in the accessibility of CRTs suggested by our survey may indicate that recent national policy campaigns in England to achieve better access to mental health crisis care^15^^,^^23^ have had some positive effect. Notwithstanding the need for more research evidence about effective service models, the current ‘postcode lottery' found by our survey regarding the availability of specialist crisis services for children and older adults indicates a need for action from policy makers and service planners to ensure appropriate services are provided in all areas for these vulnerable groups at times of crisis.

## Supplementary material

For supplementary material accompanying this paper visit http://dx.doi.org/10.1192/bjb.2018.19. click here to view supplementary material

## About the authors

**Brynmor Lloyd-Evans** is a Senior Lecturer, **Danielle Lamb** is a Senior Research Associate, **Joe Barnby, Michelle Eskinazi** and **Amelia Turner** are Research Assistants, and **Sonia Johnson** is Professor of Social and Community Psychiatry in the Division of Psychiatry at University College London, UK.
