# Supplementary material for: Mental health crisis resolution teams and crisis care systems in England: a national survey
Source: BJPsych Bull. 2018 Aug;42(4):146–51. doi: 10.1192/bjb.2018.19 (PMC6436049; doi:10.1192/bjb.2018.19)
Supplement: Supplementary file 1 [file S2056469418000190sup001.docx]

**Mental Health Crisis Resolution Teams and Crisis Care Systems in England: a national survey**

**Data Supplement DS1: Survey results - descriptive data**

**1. Team Inclusion and Age Limits (Adult CRTs only)**

**CRT eligibility criteria - age**

The table below demonstrates that most adult teams work with older adults (OAs) over the age of 65 regardless of age, however most do not work with Children and Young People (CYP) under the age of 18. Of the teams working with CYP, 22.2% (42/190) have been given specific training to work with this population. Of the teams working with OAs, 26.5% (47/179) have been given specific training to work with this population.

| **Age Range** | **Number of Teams** | **% of teams** |
| --- | --- | --- |
| Accepts 18+ | 118/190 | 62.1 |
| Accepts 17+ | 12/190 | 6.3 |
| Accepts 16+ | 42/190 | 22.1 |
| Accepts under 16s | 18/190 | 9.5 |
| Accepts over 65 (with no upper limit) | 137/190 | 72.1 |
| Accepts over 65 (with upper limit)^^[[1]](#footnote-1)^^ | 14/190 | 7.4 |
| Accepts only up to 67^^[[2]](#footnote-2)^^ | 39/190 | 20.5 |

All but 2 of the CYP teams had no lower age limit, the two which did were 11 years old and 12 years old respectively.

**CRT eligibility criteria - diagnosis**

The vast majority of adult teams accept people with Personality Disorder (PD) as primary diagnoses for admittance to their caseload, while just under half accept people with Intellectual Disabilities (ID), and very few accept people with Dementia as illustrated by the table below.

| **Adult CRTs** | | |
| --- | --- | --- |
| **Will accept people with this Primary Diagnosis** | **Number of Teams** | **% of teams** |
| Personality Disorder | 187/190 | 98.4 |
| Intellectual Disability | 94/190 | 49.5 |
| Dementia | 32/190 | 16.8 |

100% (30/30) of all older adult and dementia teams accepted dementia as a primary diagnosis. Eleven of these thirty teams were dedicated dementia services, which did not also accept people with a mental illness but not dementia.

84.2% (16/19) of older adult teams not exclusively for people with dementia accept people with personality disorder as primary diagnosis, and only 15.8% (3/19) accept people with an intellectual disability.

76.9% of CYP teams accept people with personality disorder as a primary diagnosis, and 15.8% accept people with an intellectual disability.

**2. 24 Hour Services Offered**

**Which services were offered 24 hours a day, 7 days a week by CRTs?**

The great majority of teams offered some form of 24-hour service to individuals on their caseload. We asked whether the CRT offers a 24-hour service covering referrals by phone, phone support, assessment on NHS premises, assessment of new referrals at homes, and home visits to current CRT service users. In a small number of cases, a 24-hour service was available on weekdays but not at weekends (maximum 3.7% per category); those services are not included in the table below.

190 adult teams responded to this question, 13 CYP teams, and 30 Older Adults and Dementia (OA & D) teams.

| **24 hour Service Offered** | **Number of teams with 24hr/7 days a week service (%)** |
| --- | --- |
| **Phone Referral** | Adults: 176/190 (92.6)  CYP: 5/13 (38.5)  OA & D: 5/30 (16.7) |
| **Phone Support to current CRT service users** | Adults: 173/190 (91.1)  CYP: 8/13 (61.5)  OA & D: 9/30 (30) |
| **Assessment of new referrals on NHS premises** | Adults: 161/190 (84.7)  CYP: 7/13 (53.8)  OA & D: 5/30 (16.7) |
| **Assessment of new referrals at home** | Adults: 128/190 (67.4)  CYP: 7/13 (53.8)  OA & D: 9/30 (30) |
| **Will visit current CRT service users at home** | Adults: 132/190 (69.5)  CYP: 6/13 (46.2)  OA & D: 9/30 (30) |

**3. Team Structure and Staffing**

**What are the numbers of Doctors, Nurses, Occupational Therapists, Social Workers, Psychologists, and Support Workers in each CRT?**

The following table describes the types of staff employed in adult, CYP, and OA and Dementia CRT teams.

| **Staff member** | **Teams with these staff (n)** | **%** | **Mean FTE: in teams employing this staff group** | **Mean FTE: all respondents** |
| --- | --- | --- | --- | --- |
| **Adults (n=185)** | | | | |
| Consultant psychiatrist | 163 | 88.1 | 1.1 | 1.09 |
| Other doctor | 133 | 71.9 | 1.6 | 1.11 |
| Any medical staffing (Consultants or other grade) | 173 | 93.5 |  | |
| Nursing staff | 182 | 98.4 | 14.12 | 13.6 |
| Social worker | 105 | 56.8 | 1.89 | 1.07 |
| Occupational therapist | 88 | 47.6 | 1.50 | 0.72 |
| Psychologist | 73 | 39.5 | 1.08 | 0.43 |
| Support Worker | 162 | 87.6 | 4.33 | 3.71 |
| Peer Support Worker | 9 | 4.9 | 4.18 | 0.2 |
| Teams with 5+ types of MHPs | 22 | 11.9 |  | |
| Teams with 4+ types of MHPs | 73 | 39.5 |  | |
| Teams with 3+ types of MHPs | 132 | 71.4 |  | |
| Mean fte Staff (all respondents) |  | | | 22.32 |

| **Staff member** | **Teams with this type of staff within the team**  **/13** | **%** | **Mean FTE: in teams employing this staff group** | **Mean FTE all respondents** |
| --- | --- | --- | --- | --- |
| **CYP (n=13)** | | | | |
| Consultant psychiatrist | 4 | 30.8 | 1.3 | 0.4 |
| Other doctor | 4 | 30.8 | 1.2 | 0.4 |
| Any medical staffing | 6 | 46.2 |  | |
| Nursing staff | 13 | 100 | 9.3 | 9.3 |
| Social worker | 8 | 61.5 | 1.5 | 0.9 |
| Occupational therapist | 4 | 30.8 | 1.1 | 0.3 |
| Psychologist | 2 | 15.4 | 1.0 | 0.2 |
| Support Worker | 3 | 23.1 | 2.3 | 0.5 |
| Peer Support Worker | 0 | 0 |  | |
| Teams with 5+ types of MHPs | 4 | 30.8 |  | |
| Teams with 4+ types of MHPs | 7 | 53.8 |  | |
| Teams with 3+ types of MHPs | 10 | 76.9 |  | |
| Mean fte Staff (all respondents) | 12.0 | | | |

| **Staff member** | **Teams with this type of staff within the team**  **/29** | **%** | **Mean FTE: in teams employing this staff group** | **Mean FTE all respondents** |
| --- | --- | --- | --- | --- |
| **Older Adults & Dementia (n=29)** | | | | |
| Consultant psychiatrist | 17 | 58.6 | 2.6 | 1.5 |
| Other doctor | 9 | 31.0 | 0.9 | 0.3 |
| Any medical staffing | 11 | 37.9 |  | |
| Nursing staff | 29 | 100 | 8.8 | 8.8 |
| Social worker | 2 | 6.9 | 1.0 | 0.1 |
| Occupational therapist | 16 | 55.2 | 1.0 | 0.5 |
| Psychologist | 8 | 27.6 | 0.8 | 0.2 |
| Support Worker | 25 | 86.2 | 5.0 | 4.3 |
| Peer Support Worker | 0 | 0 |  | |
| Teams with 5+ types of MHPs | 9 | 31.0 |  | |
| Teams with 4+ types of MHPs | 14 | 48.3 |  | |
| Teams with 3+ types of MHPs | 21 | 72.4 |  |  |
| Mean fte Staff (all respondents) | 15.7 | | |  |

**4. Working with inpatient services, Crisis Houses, Acute Day Units, and Crisis Cafés**

We asked teams whether they were responsible for Psychiatric Liaison Services (PLS) in their locality, whether joint assessments were made always, sometimes, rarely, or never, as well as whether there was a Crisis House, Acute Day Unit (ADU) or Crisis Café^1^[[3]](#footnote-3)^^ in their area.

**Does the CRT have Psychiatric Liaison Service responsibility?**

| **Type of Service** | **Yes** |
| --- | --- |
| Adult (n=185) | 77/185 (41.6%) |
| CYP (n=13) | 9/13 (69.2) |
| OA’s and Dementia (n=29) | 4/29 (13.8%) |

**Does the CRT conduct joint assessment with Psychiatric Liaison Services where appropriate**

| **Type of Service** |  |
| --- | --- |
| Adult | Always: 22/185 (11.9%)  Sometimes: 82/185 (44.3%)  Rarely: 39/185 (21.1%)  Never: 42/185 (22.7%) |
| CYP | Always: 02/13 (15.4%)  Sometimes: 04/13 (30.8%)  Rarely: 03/13 (23.2%)  Never: 04/13 (30.8%) |
| OA’s and Dementia (n=29) | Always: 02/29 (06.9%)  Sometimes: 08/29 (27.6%)  Rarely: 08/29 (27.6%)  Never: 11/29 (37.9%) |

**Teams with access to a Crisis House**

| **Type of CRT Service** | **Services with access (%)** | **CRT has gatekeeping responsibilities for the crisis house (%)** | **Crisis house care is supported by the CRT (%)** |
| --- | --- | --- | --- |
| Adult | 85/185 (45.9) | All beds : 40/84 (47.6)  Most: 09/84 (10.7)  Some: 14/84 (16.7)  Never: 21/84(25.0) | All service users: 47/84 (55.6)  Most: 16/84 (19.0)  Some: 19/84 (22.6)  None: 02/84 (02.4) |
| CYP | 1/13 (7.69) | All: 0  Most: 1/1 (100)  Some: 0  Never: 0 | All: 1/1 (100)  Most: 0  Some: 0  None: 0 |
| OA’s and Dementia | 3/29 (10.3) | All: 1/3 (33.3)  Most: 0  Some: 0  Never: 2/3 66.7) | All: 2/3 (66.7)  Most: 0  Some: 0  Never: 1/3 (33.3) |

**Q. Teams with access to an Acute Day Unit**

| **Type of CRT Service** | **Services with access (%)** | **CRT has Gatekeeping Responsibilities for the crisis house (%)** | **Crisis house care is supported by the CRT (%)** |
| --- | --- | --- | --- |
| Adult | 40/185 (21.6) | All SUs: 19/39 (48.7)  Most: 06/39 (15.4)  Some: 02/39 (05.1)  Never: 12/39 (30.8) | All SUs: 13/39 (33.3)  Most: 09/39 (23.1)  Some: 15/39 (38.5)  Never: 02/39 (05.1) |
| CYP | 1/13 (7.69) | All: 0  Most: 1/1 (100)  Some: 0  Never: 0 | All SUs: 0  Most: 1/1 (100)  Some: 0  Never: 0 |
| OA’s and Dementia | 5/29 (17.2) | All: 0  Most: 0  Some: 1/5 (20)  Never: 4/5 (80) | All: 0  Most: 0  Some: 4/5 (80)  Never: 1/5 (20) |

**Teams with access to a Crisis Cafe**

Adult: 28/185 (15.1%) [5/28 (17.9%) are 24 hour]

CYP: 01/13 (7.69%) [0/1 (0%) are 24 hour]

OA’s and Dementia: 03/29 (10.3) [1/29 (3.5%) are 24 hour]

**5. Gatekeeping and referrals**

**Percentage of CRT teams where CRT staff assess individuals *in person* before hospital admission**

| **Face-to-face assessment provided** | | | |
| --- | --- | --- | --- |
|  | **Adult** | **CYP** | **OA’s and Dementia** |
| Always: | 92/185 (49.7%) | 4/13 (30.8%) | 9/29 (31.0%) |
| Often: | 68/185 (36.8%) | 6/13 (46.2%) | 16/29 (55.2%) |
| Some: | 23/185 (12.4%) | 2/13 (15.4%) | 2/29 (6.9%) |
| None: | 2/185 (01.1%) | 1/13 (7.69%) | 2/29 (6.9%) |

**Q. Percentage of CRT teams where telephone discussion between CRT, and referrer and/or service user, takes place before hospital admission**

| **Phone consultation provided** | | | |
| --- | --- | --- | --- |
|  | **Adult** | **CYP** | **OA’s and Dementia** |
| Always: | 124/185 (67.0%) | 3/13 (23.1%) | 16/29 (55.2) |
| Often: | 28/185 (15.1%) | 3/13 (23.1%) | 8/29 (27.6) |
| Some: | 16/185 (08.6%) | 5/13 (38.5%) | 3/29 (10.3) |
| None: | 17/185 (09.2%) | 2/13 (15.4%) | 2/29 (6.9%) |

**CRT attendance at Mental Health Act Assessments**

| **Does a member of the CRT team attend MHA assessments** | | | |
| --- | --- | --- | --- |
|  | **Adult** | **CYP** | **OA’s and Dementia** |
| Always: | 35/185 (18.9%) | 3/13 (23.1%) | 2/29 (6.9%) |
| Often: | 47/185 (25.4%) | 3/13 (23.1%) | 10/20 (34.5) |
| Some: | 92/185 (49.7%) | 6/13 (46.2%) | 15/29 (51.7%) |
| None: | 11/185 (05.9%) | 1/13 (7.69%) | 2/29 (6.9%) |

**Is there a separate assessment team that triages service users in person before acceptance to the crisis team caseload?**

|  | **Yes (%)** | **If yes, does this accept referrals 24 hours a day?** |
| --- | --- | --- |
| **Adults:** | 59/184 (32.1%) | 42/59 (71.2%) |
| **CYP:** | 2/13 (15.4%) | 1/2 (50%) |
| **OA&D:** | 6/29 (20.7%) | 3/6 (50%) |

**Crisis line staff and authority to refer**

We asked whether there was a (separate, publically accessible) crisis line phone service that acted as a first point of contact for crisis referrals. 184 CRTs answered the initial question.

% of CRT teams where a crisis line is used

**Adult** 132/184 (71.7 %)

**CYP** 7/13 (53.8%)

**OA & Dementia** 16/29 (55.2%)

% of CRT teams where the crisis line operates 24 hours (out of CRT teams who have reported that there is one locally)

**Adult** 106/132 (80.3%)

**CYP** 3/7 (42.9%)

**OA & Dementia**  10/16 (62.5%)

% of CRT teams where there is a crisis line whose manager also manages the crisis line

**Adult** 54/131 (41.2%)

**CYP** 2/7 (28.6%)

**OA & Dementia** 3/16 (18.8%)

% of CRT teams where there is a crisis line whose staff also staff the crisis line

**Adult** 70/130 (53.8%)

**CYP** 5/7 (71.4%)

**OA & Dementia** 7/16 (43.8%)

% of CRT teams where there is a crisis line, where the crisis line has the authority to decide upon which service should then assess/see people in crisis

**Adult** 102/131 (77.9%)

**CYP** 5/7 (71.4%)

**OA & Dementia** 10/12 (83.3%)

% of CRT teams with access to a crisis line, where crisis line is staffed by qualified Mental Health Professionals

**Adult**

All: 8/131 (06.1%)

Some: 67/131 (51.1%)

None: 56/131 (42.7%)

**CYP**

All: 3/7 (42.9%)

Some: 2/7 (28.6%)

None: 0

**OA & Dementia**

All: 10/16 (62.5%)

Some: 6/16 (37.5%)

None: 0

**Referral sources**

All respondents (n=184) reported that they accept referrals from other mental health services. We asked whether they also accepted self-referrals, referrals from GPs, and referrals from third sector organisations (e.g. day centres).

Most teams accept referrals from GPs and other community professionals. Just over half accept self-referrals from clients whom they already know, but most do not accept referrals from clients whom they don’t know.

| **CRT will accept referrals from this source** | **Adult/n=184**  **(%)** | **CYP/ n=13**  **(%)** | **OA & Dementia/ n=29 (%)** |
| --- | --- | --- | --- |
| GP | 148/184 (80.4) | 9/13 (69.2) | 22/29 (75.9) |
| Other primary care clinicians e.g. practice nurse | 133/184 (72.3) | 7/13 (53.8) | 17/29 (58.6) |
| Secondary mental health services | 183/184 (99.5) | 12/13 (92.3) | 28/29 (96.6) |
| NHS 111 service | 108/184 (58.7) | 6/13 (46.2) | 11/29 (37.9) |
| Police | 132/184 (71.7) | 9/13 (69.2) | 13/29 (44.8) |
| IAPT | 132/184 (71.7) | 7/13 (53.8) | 13/29 (44.8) |
| Other emergency services | 116/184 (63.0) | 10/13 (76.9) | 13/29 (44.8) |
| Psychiatric Liaison | 180/184 (97.8) | 9/13 (69.2) | 29/29 (100) |
| Self-referral (Service User not known to the CRT service) | 79/184 (42.9) | 5/13 (38.5) | 7/29 (24.1) |
| Self-referral (Service User known to service) | 127/184 (67.4) | 6/13 (46.2) | 13/29 (44.8) |
| Third sector organisations | 91/184 (49.5) | 6/13 (46.2) | 9/29 (31.0) |
| Other | 40/184 (21.7) | 5/13 (38.5) | 7/29 (24.1) |

**6. Caseload and staffing**

**Mean, standard deviation (*SD*), and range of CRT team caseloads, and average staffing per 25 and 30 cases**

180 teams answered the question about their current caseload and 173 teams answered the question about their typical lower and upper ranges of their caseload. From this, the mean staff per 25 and 30 cases across adult, CYP, and OA &Dementia teams were calculated.

| **Adult** | **Mean fte (*SD*)** | ***N*** | **Range** |
| --- | --- | --- | --- |
| Current caseload | 35.4 (*23.6*) | (N = 181) | 139 (5-144) |
| Lowest typical caseload | 24.8 (*18.7*) | (N = 178) | 120 (0-120) |
| Highest typical caseload | 47.2 (*27.8*) | (N = 173) | 180 (10-190) |

DH guidelines from 2001^15^, recommend that that a CRT team for working age adults should consist of no fewer than 14 fte staff for a caseload of 25-30 service users.

A table showing how many teams have at least 14 fte staff per 25 and 30 cases, both for their current reported caseload and typical highest caseload, is reported below. While a majority of adult CRTs achieved recommended staffing levels for their current caseload, only about half of teams did so for their maximum typical caseload.

| **Adult** | **Current reported caseload** | **Highest typical caseload** |
| --- | --- | --- |
| At least 14 Staff/ 25 cases | 118/ 180 (65.5%) | 71/ 172 (41.3%) |
| At least 14 Staff/ 30 cases | 137/ 180 (76.1%) | 95/ 172 (55.2%) |
| Mean Ratio (Staff: caseload) | 0.82 | 0.56 |

| **CYP** | **Mean fte (*SD*)** | ***N*** | **Range** |
| --- | --- | --- | --- |
| Current caseload | 22.42 *(15.3*) | (N=12) | 46 (3-49) |
| Lowest typical caseload | 16.40 *(11.6)* | (N=10) | 30 (0-30) |
| Highest typical caseload | 33.83 *(22.9)* | (N=12) | 74 (6-80) |

| **CYP** | **Current reported caseload** | **Highest typical caseload** |
| --- | --- | --- |
| At least 14 Staff/ 25 cases | 6/ 12 (50%) | 4/ 12 (33.3%) |
| At least 14 Staff/ 30 cases | 7/ 12 (58.3%) | 5/ 12 (41.6%) |
| Mean Ratio (Staff: caseload) | 1.05 | 0.66 |

| **OA & Dementia** | **Mean fte (*SD*)** | ***N*** | **Range** |
| --- | --- | --- | --- |
| Current caseload | 35.0 *(42.1)* | (N=29) | 218 (8-226) |
| Lowest typical caseload | 26.3 *(37.7)* | (N=28) | 196 (4-200) |
| Highest typical caseload | 50.6 *(64.3)* | (N=27) | 335 (15-350) |

| **OA & Dementia** | **Current reported caseload** | **Highest typical caseload** |
| --- | --- | --- |
| At least 14 Staff/ 25 cases | 13/29 (44.8%) | 7/27 (25.9%) |
| At least 14 Staff/ 30 cases | 17/29 (58.6%) | 10/27 (37.0%) |
| Mean Ratio (Staff: caseload) | 0.63 | 0.45 |

**7. Referral response times, minimum care, and clinical measures**

**Response times**

184 teams answered this question and reported any targets for response times after receiving a referral. This question asked teams to note whether they had a specific target time in which to respond with an initial decision about planned response, start an assessment in person with a service user, and complete an assessment.

Most teams reported a target response time for each category, with specific targets for commencing an assessment being most widely reported. The percentage of teams with specific targets, the range and median target time among teams with a target, and the number of teams with response time targets of 4 hours or less are reported. [NB: the figures reported here are the number of CRT teams which set response time targets. We did not collect data about to what extent these targets are met.]

| **Teams which have a target response time to…** | **Number of teams responding with a specific target**  **(%)** | **Median target response time**  **(hrs)** | **Range of target response times**  **(hrs)** | **% CRTs which set a target of 4hrs or less response time** |
| --- | --- | --- | --- | --- |
| **Adult** | | | | |
| **respond to** a referral: | 128/184 (69.5) | 04 | 0-48 | 55.7  (n=128) |
| **commence** an assessment: | 159/184 (86.4) | 04 | 1-168 | 45.4  (n=159) |
| **complete** an assessment: | 118/184 (64.1) | 24 | 3-672 | 20.0  (n=118) |
| **CYP** | | | | |
| **respond to** a referral: | 8/13 (61.5) | 2.5 | 0.25-24.00 | 87.5  (n=8) |
| **commence** an assessment: | 11/13 (84.6) | 04 | 1-196 | 63.6  (n=11) |
| **complete** an assessment: | 6/13 (46.2) | 24 | 4-244 | 20  (n=5) |
| **OA & Dementia** | | | | |
| **respond to** a referral: | 20/29 (69.0) | 04 | 2-48 | 70  (n=20) |
| **commence** an assessment: | 18/29 (62.1) | 14 | 4-120 | 33.3  (n=15) |
| **complete** an assessment: | 16/29 (55.2) | 24 | 4-120 | 15.4  (n=13) |

**Duration of care**

We asked whether there was a limit to the duration of care for CRT service users, whether there was a minimum number of visits given per week to each individual on the caseload, and whether a minimum duration of time was stipulated for each visit.

Although teams gave a limit to the amount of time they would see a service user, this was most often reported to be flexible based upon the presentation of each individual, and therefore the following figures should represent guidelines that each team abides by, as opposed to solid and rigid protocol.

The table below reports data from respondents who provided one figure for the minimum number of visits per week per service user. CRT teams which categorise service users by risk or needs (e.g. using a “traffic lights” or “RAG” system)and have different expectations for the frequency of visits to service users in each category, are not included in this table.

|  | **Number of teams (%)** | **Median** |
| --- | --- | --- |
| **Adult** | | |
| Limit to time on caseload (days): | 58/184 (31.5) | 42 days |
| Minimum number of visits given/ week: | 48/175 (28.2) | 2 per week |
| Minimum time given per visit/ minutes: | 28/184 (15.2) | 60 minutes |
| **CYP** | | |
| Limit to time on caseload (days): | 5/13 (38.5) | 14 days |
| Minimum number of visits given/ week: | 3/13 (23.1) | 2 per week |
| Minimum time given per visit/ minutes: | 1/13 (7.7) | 60 minutes |
| **OA & Dementia** | | |
| Limit to time on caseload (days): | 17/29 (58.6) | 44 days |
| Minimum number of visits given/ week: | 7/29 (24.1) | 3 days per week |
| Minimum time given per visit/ minutes: | 1/29 (3.4) | 30 minutes |

**Routine measurement of clinical outcomes and service user experience**

Teams were asked whether they routinely measured clinical outcomes or service user experience, and whether teams used a particular philosophy of care.

Results suggest that in adult teams, most employ measures to assess service user clinical outcomes and experience, such as HONOS and the Friends and Family Test respectively.

| **Number of teams in which:** |  |
| --- | --- |
| **Adult** | |
| **Clinical outcome** measures are used routinely: | 137/184 (74.6%) |
| **User experience** measures are used routinely: | 168/184 (91.0%) |
| **CYP** | |
| **Clinical outcome** measures are used routinely: | 10/13 (76.9%) |
| **User experience** measures are used routinely: | 13/13 (100%) |
| **OA & Dementia** | |
| **Clinical outcome** measures are used routinely: | 19/29 (65.5%) |
| **User experience** measures are used routinely: | 23/29 (79.3%) |

**Theoretical models and philosophy of care**

**Adults:**

81 of the 183 teams (44.3%) that responded to this question stated that they do follow a specific philosophy of care or theoretical model within their team.

Within these 81 teams, the ‘recovery model’ was the approach most explicitly reported (25/81), with other teams also referencing aspect of their care which covered aspects of the recovery model e.g. compassionate care, but did not explicitly mention the recovery model.

The next most common responses were the “biopsychosocial” (n=10) and “CORE CRT crisis model” (n=10). Other approaches that were reported were “client centred”, “HTAS”, and “FACT” models.

**CYP:**

Only 3 of the 13 CYP teams that responded to this question stated a specific philosophy of care within their team which included ‘psychosocial’ and a ‘DBT model.’

**OA & Dementia:**

15 of the 29 teams that responded to this question stated that they do follow a specific philosophy of care within their team. Four teams reported using variations on a ‘recovery model’ and other single teams mentioned the following: ‘social systems’; ‘Stokes model with organic patients’, ‘patient centred care’ and the ‘Ian James model of psychological care.’

**8. Team training**

**Team training and frequency of training**

Most CRT teams did not say that they provided their staff with regular additional CRT-specific training in addition to mandatory NHS Trust training. Where specific CRT training was provided, this was typically less frequent than every two months.

| **Type of Service** | **Services that provide specific CRT team training* (%)** | **Frequency of training (%)** |
| --- | --- | --- |
| Adult | 76/183 (41.5) | Less than every two months: 38/76 (50.0)  At least every two months: 15/76 (19.7)  At least every month: 23/76 (30.3) |
| CYP | 4/13 (30.8) | Less than every two months: 02/04 (50%)  At least every two months: 01/04 (25%)  At least every month: 01/04 (25%) |
| OAs and Dementia | 8/29 (27.6) | Less than every two months: 05/08 (63%)  At least every two months: 01/08 (12%)  At least every month: 02/08 (25%) |

*Separate/additional to generic NHS trust training for CRT staff

**9. Associated services**

**Other crisis services (reported by adult CRT teams).**

Adult CRT managers were asked to identify any local crisis services for older adults, CYP or people with substance misuse problems. Their responses are reported below.

[NB: All OA and CYP crisis services identified by adult CRT managers were contacted as part of the mapping for this survey. It transpires that most of these services were community teams also providing longer term support to service users: only stand-alone CRT teams for older adults and CYPs have been included elsewhere in this survey.]

**Local Specialist Crisis Services Identified by the Adult CRT Teams’ Survey Responses**

| **Type of specialist crisis service** | **Adult CRTs which reported a separate specialist service in the area**  **(%)** | **Adult CRTs reported separate specialist services that operate 24hrs a day**  **(%)** | **Age and diagnosis limitations applying to separate, specialist crisis service**  **(%)** |
| --- | --- | --- | --- |
| CYP | 91/182  (50.0) | 23/91  (25.3) | Accepts all ages 0-18:  44/91 (48.4) |
| OA & Dementia | 77/183  (42.1) | 11/77  (14.3) | Works just with OA with mental illness:  52/67 (77.6)  Works just with adults with Dementia:  03/67 (04.5)  Works with both OA with mental illness and adults with Dementia:  01/67 (01.5) |
| Substance Abuse | 21/182  (11.5) |  | |

1. Some teams imposed restrictions upon their OA caseload. This was variable between teams, and ranged from allowing individuals who were previously on their adult caseload to having an upper age cap of 75. [↑](#footnote-ref-1)
2. Teams which only worked with people for up to 2 years beyond the current retirement age of 65 were included here, as services which essentially cater only for working age adults. [↑](#footnote-ref-2)
3. Crisis cafes offer support and a safe place for people in a crisis outside of normal office hours and are intended to offer an alternative to attending A&E where possible. [↑](#footnote-ref-3)
